# Pan-cancer analysis reveals DDX21 as a potential biomarker for the prognosis of multiple tumor types

**DOI:** 10.3389/fonc.2022.947054

**Published:** 2022-11-24

**Authors:** Ankang Hu, Yonghui Wang, Jiahao Tian, Zihan Chen, Renjin Chen, Xufeng Han, Yang Chen, Tingjun Liu, Quangang Chen

**Affiliations:** ^1^ Laboratory Animal Center, Xuzhou Medical University, Xuzhou, Jiangsu, China; ^2^ School of Life Science, Xuzhou Medical University, Xuzhou, Jiangsu, China; ^3^ Clinical Medicine Science, The Affiliated Hospital of Xuzhou Medical University, Xuzhou, Jiangsu, China; ^4^ Cancer Institute, Xuzhou Medical University, Xuzhou, Jiangsu, China

**Keywords:** DDX21, biomarker, pan-cancer, prognosis, immune infiltration

## Abstract

**Background:**

DExD-box helicase 21 (DDX21) is an essential member of the RNA helicase family. DDX21 is involved in the carcinogenesis of various malignancies, but there has been no comprehensive research on its involvement in different types of cancer.

**Method:**

This study used TCGA, CPTAC, GTEx, GEO, FANTOM5, BioGRID, TIMER2, GEPIA2, cBioPortal, STRING, and Metascape databases and Survival ROC software to evaluate DDX21 gene expression, protein expression, immunohistochemistry, gene mutation, immune infiltration, and protein phosphorylation in 33 TCGA tumor types, as well as the prognostic relationship between DDX21 and different tumors, by survival analysis and similar gene enrichment analysis. Furthermore, Cell Counting Kit-8 (CCK-8) and Transwell studies were employed to assess the effect of DDX21 expression on lung adenocarcinoma (LUAD) cell proliferation and migration.

**Result:**

The DDX21 gene was highly expressed in most cancers, and overexpression was associated with poor overall survival (OS) and disease-free survival (DFS). DDX21 mutations were most common in uterine corpus endometrial carcinoma (UCEC; >5%), and DDX21 expression was positively correlated with the degree of infiltration of CAF and CD8^+^ cells in several tumor types. Numerous genes were co-expressed with DDX21. Gene enrichment analysis revealed close links between DDX21, RNA metabolism, and ribosomal protein production. *In vitro* analysis of LUAD cells showed that DDX21 expression was positively correlated with cell proliferation and migration capacity, consistent with prior bioinformatics studies.

**Conclusions:**

DDX21 is overexpressed in a variety of cancers, and overexpression in some cancers is associated with poor prognosis. Immune infiltration and DDX21-related gene enrichment analyses indicated that DDX21 may affect cancer development through mechanisms that regulate tumor immunity, RNA metabolism, and ribosomal protein synthesis. This pan-cancer study revealed the prognostic value and the oncogenic role of DDX21.

## Introduction

Given the complexities of tumorigenesis, it is critical to perform a pan-cancer expression study of any gene of interest, analyze its relationship to clinical prognosis, and explore possible molecular mechanisms. Various publicly funded databases, including The Cancer Genome Atlas (TCGA), Clinical Proteomic Tumor Analysis Consortium (CPTAC), Genotype-Tissue Expression (GTEx), Gene Expression Omnibus (GEO), Human Protein Atlas (HPA), FANTOM5 (Function ANnoTation Of the Mammalian genome), and the Biological General Repository for Interaction Datasets (BioGRID), contain functional genomics datasets for different tumor types (1–3), which facilitates pan-cancer analyses.

DExD-box helicase 21 (DDX21) proteins, characterized by the conserved Asp-Glu-Ala-Asp (DEAD) motif, are predicted RNA helicases involved in a variety of cellular processes including RNA secondary structure alterations, translation initiation, nuclear and mitochondrial splicing, and ribosome and spliceosome assembly (4, 5). Numerous members of this group are thought to play a role in embryogenesis, spermatogenesis, and cellular development and division, based on their distribution patterns. In individuals with watermelon stomach illness, autoimmune antibodies recognize the DEAD box pattern as an antigen. DDX21 untangles double-stranded RNA and folds single-stranded RNA. It may also be involved in ribosomal RNA synthesis, RNA editing, RNA transport, and general transcription (6, 7). Nevertheless, research into the role of DDX21 in tumors has largely been restricted to specificcancer types, such as breast cancer and ACC (adrenocortical carcinoma) (8–10), and no pan-cancer analysis of the link between DDX21 and other cancers has been performed.

Herein, we conducted a pan-cancer investigation of DDX21 using TCGA, GEO, GTEx, CPTAC, HPA, FANTOM5, and BioGRID datasets. To study the probable molecular pathways of DDX21 in the etiology and clinical prognosis of various malignancies, we also incorporated gene expression, genetic alteration, immunohistochemistry staining, survival status, immune infiltration, and gene set enrichment analysis data.

## Materials and methods

### Gene expression analysis

We loaded DDX21 into the “Gene DE” module of the tumor immune estimation resource, version 2 (TIMER2; http://timer.cistrome.org/), and compared the DDX21 expression between tumor and nearby normal tissues in TCGA for different tumors and particular tumor subtypes (workflow diagram, **Supplementary Figure S1** **)**. For certain tumors without normal tissues or with limited normal tissues (e.g., TCGA-ACC for adrenocortical carcinoma and TCGA-OV for ovarian serous cystadenocarcinoma), the “Expression analysis-Expression DIY-Box Plots” module of the gene expression profiling interactive analysis version 2 (GEPIA2) web server (http://gepia2.cancer-pku.cn/#analysis) (11) was used to generate box plots of expression differences between these tumor tissues and corresponding normal tissues in the GTEx database, with p-value cutoff = 0.01, log2 fold change cutoff = 1, and “Match TCGA normal and GTEx data” selected (workflow diagram, **Supplementary Figure S1** **)**.

### Protein expression analysis

The UALCAN portal (http://ualcan.path.uab.edu/analysis-prot.html), an interactive web resource for analyzing cancer Omics data, allowed us to conduct protein expression analysis of the CPTAC dataset (12). Herein, we explored the abundance of the DDX21 protein (NP_004719.2) in primary tumors and normal tissues by selecting “DDX21.” Available datasets for six tumors were selected, including lung adenocarcinoma (LUAD), ovarian cancer, uterine corpus endometrial carcinoma (UCEC), colon cancer, breast cancer, and clear cell renal cell carcinoma (RCC) (workflow diagram, **Supplementary Figure S1** **)**.

Immunohistochemistry (IHC) staining data from the evaluation of differences in DDX21 expression at the protein level and IHC images of DDX21 protein levels in seven tumor tissues and normal tissues, including ovarian serous cystadenocarcinoma (OV), lung squamous cell carcinoma (LUSC), rectum adenocarcinoma (READ), cervical squamous cell carcinoma and endocervical adenocarcinoma (CESC), LUAD, colon adenocarcinoma (COAD), breast invasive carcinoma (BRCA), head and neck squamous cell carcinoma (HNSC), and liver hepatocellular carcinoma (LIHC), were downloaded from the “PROTEIN EXPRESSION” part of the “PATHOLOGY” module in the HPA (http://www.proteinatlas.org/) and analyzed (workflow diagram, **Supplementary Figure S2** **)**.

Using the HPA database (version 20.1; https://www.proteinatlas.org/), we also created a DDX21 protein and RNA expression graph in the “TISSUE” module after entering “DDX21.” (13). At the same time, we also made a single-cell-type expression map of DDX21 in the “SINGLE CELL” part (workflow diagram, **Supplementary Figure S2** **)**.

### Survival prognosis analysis

We determined overall survival (OS) and disease-free survival (DFS) image data for DDX21 throughout all TCGA tumors using the GEPIA2 Survival Map module (11). To distinguish high- and low-expression cohorts, cutoff-high (50%) and cutoff-low (50%) values were used as expression criteria. Log-rank analysis was applied in the hypothesis test, and survival maps were created using the GEPIA2 Survival Analysis module (Workflow diagram, **Supplementary Figure S1**).

TCGA and GTEx data were then retrieved, and a receiver operating characteristics (ROC) curve was generated using the “Survival ROC” software tool. The abscissa and vertical axes of the ROC curve graph represent false-positive and true-positive ratios, respectively. Better prognosis accuracy is shown by a bigger area under the ROC curve (AUC) (**Supplementary Table S1**).

### Genetic alteration analysis

After inputting into the cBioPortal site (https://www.cbioportal.org/) (14, 15), we selected the “TCGA Pan Cancer Atlas Studies” part in the “Quick Select” area and typed in “DDX21” in the “Query By Gene” module for questions about the genetic alteration features of DDX21. The “Cancer Types Summary” module displayed the results for alteration frequency, mutation type, and copy number alteration (CAN) across all TCGA tumors. The “Plots” module displayed the findings of mRNA expression and RSEM (batch normalized from Illumina HiSeq RNASeqV2) throughout all TCGA tumors. Mutated site information for DDX21 may be presented in a protein structure schematic diagram using the “Mutations” module (workflow diagram, **Supplementary Figure S2**).

### Immune infiltration analysis

We used the TIMER2 web server’s “Immune-Gene” module to investigate the relationship between DDX21 expression and immune infiltration in all TCGA tumors. In terms of immune cells, CD8^+^ T-cells and cancer-associated fibroblasts were chosen. Immune infiltration was estimated using the TIMER, CIBERSORT, CIBERSORT-ABS, QUANTISEQ, XCELL, MCPCOUNTER, and EPIC algorithms. The purity-adjusted Spearman’s rank correlation test was applied to calculate the p-values and partial correlation (cor) values. A heatmap and a scatter plot were used to visualize the data (workflow diagram, **Supplementary Figure S1**).

### DDX21-related gene enrichment analysis

The STRING program (version 11.0b; https://string-db.org/) was used to generate a *Homo sapiens* DDX21 co-expression network with the following primary parameters: (1) co-expression = active interaction sources; (2) network edge meaning = evidence; (3) maximum number of interactors = 50; and (4) minimum necessary interaction score = low confidence (0.150). Then, the “Exports” module was used to export the image (workflow diagram, **Supplementary Figure S2**).

The GEPIA2 “Similar Gene Detection” module was used to extract 100 DDX21-correlated genes from the most similar transcription factors to DDX21 from TCGA datasets (workflow diagram, **Supplementary Figure S1** **)**. In Metascape (http://metascape.org), the gene characteristics of these 100 genes were used as input gene symbols for a Gene Ontology (GO) pathway enrichment study. Furthermore, pairwise gene regression analysis was performed using the GEPIA2 “Correlation Analysis” module (workflow diagram, **Supplementary Figure S2**).

### DDX21-protein interaction analysis

BioGRID’s “Network” module (version: 4.3; https://thebiogrid.org/) was used to generate a DDX21–protein interaction network, with the layout set to “Concentric Circles” (workflow diagram, **Supplementary Figure S2**).

### Cell culture

The A549 and H1299 LUAD cell line was obtained from the cell bank of Shanghai Institutes for Biological Sciences, Chinese Academy of Sciences. Cells were cultured in a DMEM high-glucose medium (KeyGEN, China) with 10% fetal bovine serum (FBS; WISENT, Canada) at 37°C with 5% CO_2_ (Thermo Scientific, USA). The DDX21 gene was amplified by PCR and subcloned into the pCDH513B lentiviral vector to construct a DDX21 overexpression plasmid. The virus was packaged in 293T cells, and after 48 h, a virus solution was used to infect A549 and H1299 cells. To suppress the expression of DDX21 using small interfering RNA (siRNA), cells were grown to 30%–50% confluency and transfected with siRNA targeting DDX21 or a non-specific control (NC) for 48 h. All transfections were performed using siLentFect according to the manufacturer’s instructions. siRNAs were synthesized in Genepharm, the sequences of which are listed in **Supplementary Table S2A**.

### Western blot

Cells were lysed in buffer containing NP-40. Equal amounts of protein were separated by SDS-PAGE. After incubation with the indicated antibodies, the immune complexes on the membrane were detected by an ECL kit (Proteintech, # PK10002). The following antibodies were acquired from commercial sources: mouse anti-DDX21 (Proteintech, #10528-1-AP) and mouse anti-Beta Actin (Proteintech, #66009-1-Ig).

### Immunofluorescence staining

Cells grown on coverslips in 24-well plates were fixed with 4% paraformaldehyde for 20 min and treated with 0.2% Triton X-100 solution for 20 min. Samples were blocked with 2% bovine serum albumin (BSA) for 1 h, followed by antibody incubation overnight at 4°C. Cells were washed three times for 5 min each time in phosphate-buffered saline (PBS) and incubated with a fluorescent-labeled secondary antibody for 90 min. After three more washes for 5 min each time in PBS, DAPI was used to stain nuclei for 15 min. A fluorescence quencher was added dropwise, coverslips were placed on slides, and edges were sealed with transparent nail polish. Images were captured by an Axio Observer confocal microscope.

### Cell proliferation assays

A549 and H1299 cells were infected with 513B-DDX21 virus or transfected with siRNA targeting DDX21 for 24 h, and then cells were seeded in 96-well plates (5 × 10^3^ cells/well) and cultured in an incubator for 0, 24, 48, or 72 h. After discarding the medium, 100 μl medium and 10 μl CCK-8 (VICMED, VC5001L-2500) were mixed in a 1.5 ml EP tube, and the prepared CCK-8 solution was added to the culture plate. After incubation at 37°C for 1 h, the optical density at 450 nm (OD_450_) was measured by a multi-plate reader (BioTek, USA).

### Transwell migration assays

Cells (4 × 10^4^) were cultured in a serum-free medium in the upper 24-well Transwell chamber, and an 800 μl culture medium (20% FBS) was added to the lower chamber. The plate was incubated at 37°C for 24 or 48 h. Finally, cells on the lower surface of the membrane were dyed to analyze cell migration. Five fields of view were randomly selected under the microscope, and images were captured and assessed using ImageJ.

### Statistical analysis

We used Prism version 8.0 (GraphPad Software, Inc.) to analyze all data in this study. Unpaired t-tests were used to compare two groups, one-way analysis of variance (ANOVA) was used to compare multiple groups, and *p* < 0.05 was considered statistically significant. All experiments were repeated at least three times.

## Results

### Gene expression analysis

**Figure 1:**
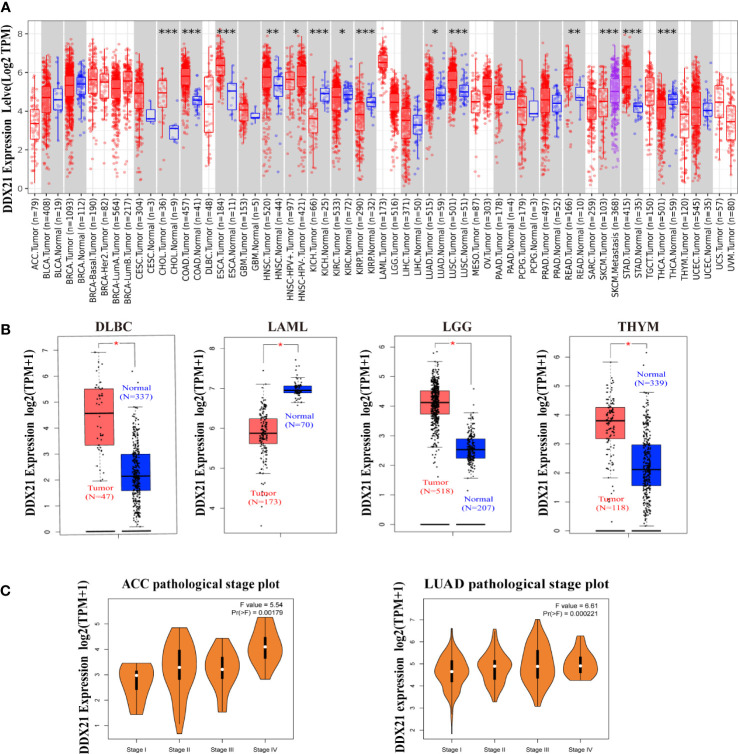
Expression levels of the DDX21 gene in different tumors and pathological stages. **(A)** Expression status of the DDX21 gene in different cancers or specific cancer subtypes analyzed by TIMER2 (*p < 0.05; **p < 0.01; ***p <0.001). **(B)** For DLBC, LAML, LGG, and THYM in TCGA, the corresponding normal tissues in the GTEx database were included as controls. Boxplot data were supplied (*p <0.01). **(C)** Based on TCGA data, expression levels of the DDX21 gene were analyzed based on the main pathological stages (I, II, III, and IV) of ACC and LUAD. Log2 (TPM + 1) was applied for log scaling.

The TIMER2 tool was used to examine the DDX21 expression status across diverse cancer types in TCGA database. As shown in **Figure 1A**, DDX21 expression levels in the tumor tissues of CHOL (cholangiocarcinoma), COAD (colon adenocarcinoma), LUSC (lung squamous cell carcinoma), ESCA (esophageal carcinoma), STAD (stomach adenocarcinoma), HNSC (head and neck squamous cell carcinoma), READ (rectum adenocarcinoma), and LUAD (lung adenocarcinoma) were higher than the corresponding control tissues. However, DDX21 expression was dramatically reduced in KICH (kidney chromophobe), KIRP (kidney renal papillary cell carcinoma), and KIRC (kidney renal clear cell carcinoma).

Using normal tissues from the GTEx dataset as controls, we further investigated expression differences in DDX21 between normal and tumor tissues for THYM (thymoma), LAML (acute myeloid leukemia), DLBC (lymphoid neoplasm diffuse large B-cell lymphoma), and LGG (brain lower grade glioma; all p < 0.05; **Figure 1B**). Other tumors such as ACC, BRAC (breast-invasive carcinoma), OV (ovarian serous cystadenocarcinoma), SARC (sarcoma), TGCT (testicular germ cell tumors), and UCS (uterine carcinosarcoma; **Supplementary Figure S3**) did not show a significant difference. GEPIA2 was also used to study the relationship between DDX21 expression and tumor pathological stages. High expression of DDX21 was shown to be substantially associated with the advanced stages of ACC and LUAD (**Figure 1C**).

### Protein expression analysis

**Figure 2:**
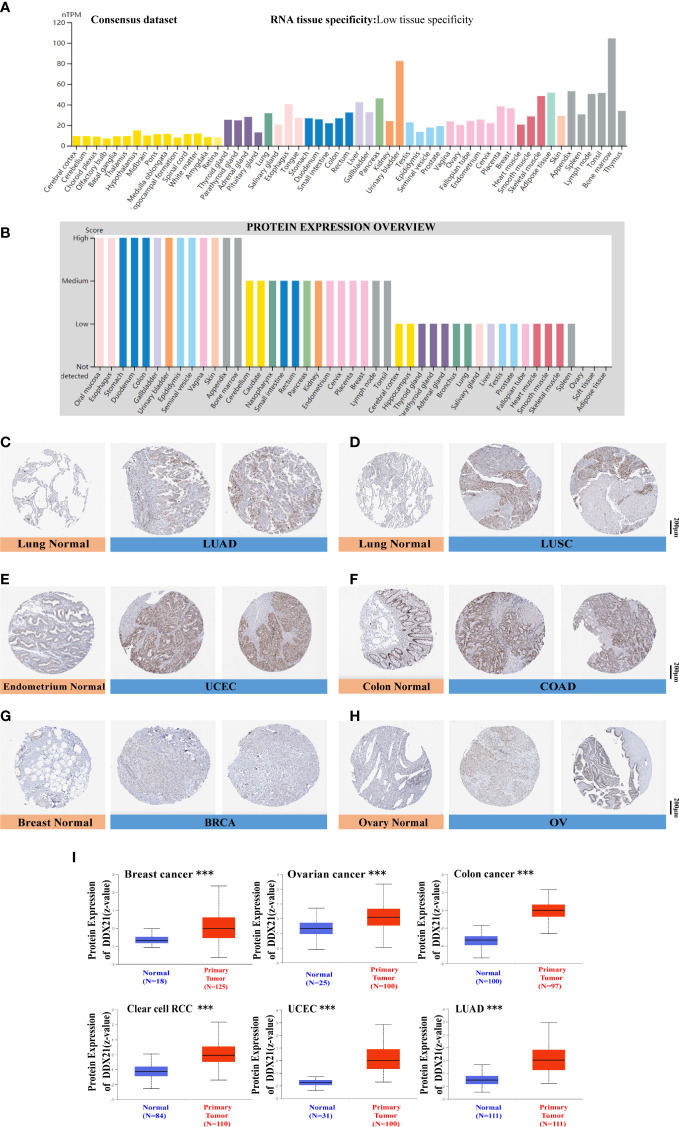
DDX21 expression profiles in human normal and cancer tissues. **(A)** Consensus DDX21 tissue expression based on datasets of HPA (Human Protein Atlas), GTEx, and FANTOM5 (Function ANnoTation Of the Mammalian genome). **(B)** Abundance of the DDX21 protein in human normal tissues. Representative IHC images of DDX21 expression between normal (left) and tumor (right) tissues. **(C, D)** Lung. **(E)** Endometrium. **(F)** Colon. **(G)** Breast. **(H)** Ovary. **(I)** Based on the CPTAC dataset, we also analyzed the abundance of DDX21 protein between normal tissues and primary breast cancer, ovarian cancer, colon cancer, clear cell RCC, UCEC, and LUAD tissues (***p < 0.001).

Based on the datasets of HPA, GTEx, and FANTOM5 (Function ANnoTation Of the Mammalian genome), we found that DDX21 was highly expressed in the lymphoid tissue, such as the appendix and bone, and enriched in the urinary bladder, the adipose tissue, and the skeletal muscle (**Figure 2A** **;** **Supplementary Figures S4A-C**). We then examined the DDX21 protein expression and found it widely expressed and at high levels in various normal tissues (**Figure 2B**). Moreover, based on single-cell RNA-seq, high expression of DDX21 was also observed in basal keratinocytes and suprabasal keratinocytes (**Supplementary Figure S4D**). Moreover, an overview of all tissues with a single-cell-type expression has been analyzed (**Supplementary Figure S5**). According to these findings, DDX21 expression has a low tissue specificity.

To evaluate DDX21 expression at the protein level, we analyzed IHC results from the HPA database and compared them with DDX21 gene expression data from TCGA. As can be seen in **Figures 2C-H**, data from the two databases were consistent. DDX21 IHC staining was mild in normal lung and endometrium tissues but high in tumor tissues. DDX21 staining was mild in normal colon and breast tissue samples but robust in tumor tissues. Normal ovary tissues had no DDX21 staining, whereas OV had moderate DDX21 staining. The CPTAC data showed higher DDX21 protein levels in primary breast cancer, ovarian cancer, colon cancer, clear cell RCC, UCEC, and LUAD tissues (**Figure 2I**, p < 0.001) than in normal tissues.

### Prognostic value of DDX21 across cancers

**Figure 3:**
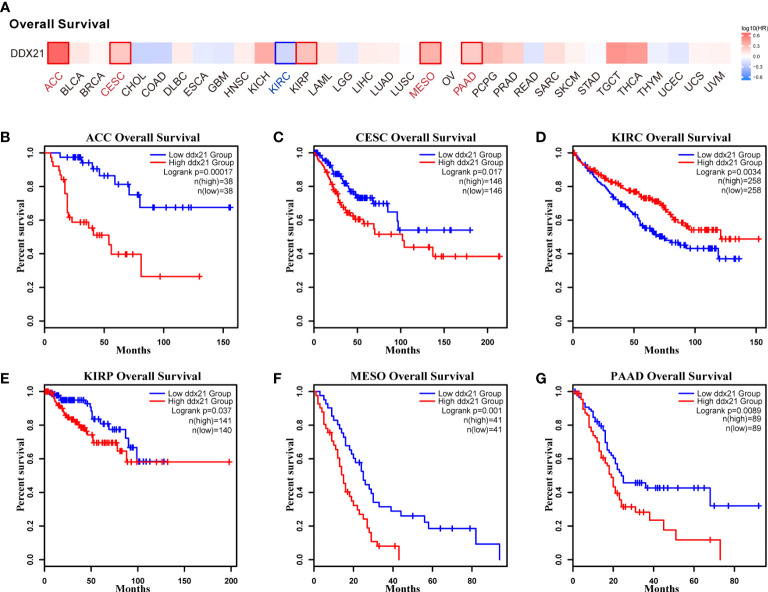
Correlation between DDX21 expression and overall survival (OS) in patients with different TCGA tumor types. GEPIA2 was used to build a survival map **(A)** and conduct overall survival analyses **(B–G)**. The survival map and Kaplan–Meier plots with significant results are displayed. The 95% confidence intervals of OS are indicated by red and blue dotted lines for high and low DDX21 expression groups, respectively.

**Figure 4:**
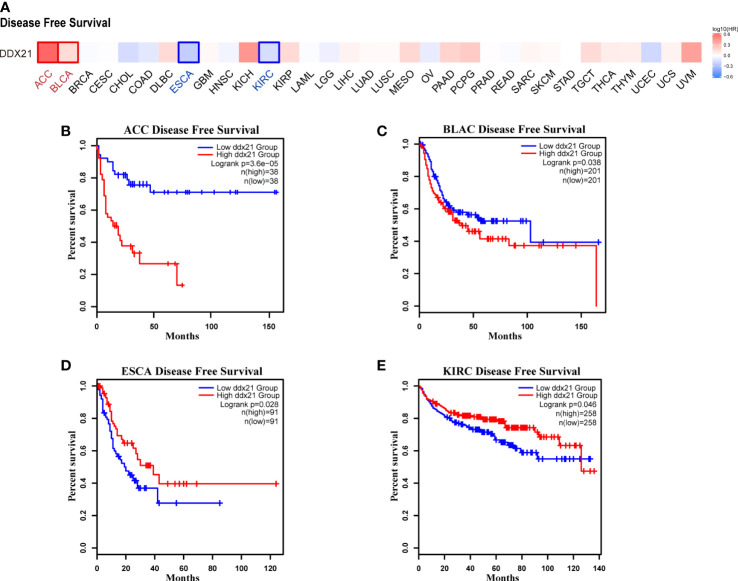
Correlation between DDX21 expression and disease-free survival (DFS) in patients with different TCGA tumor types. GEPIA2 was used to build a survival map **(A)** and conduct DFS **(B–E)** analyses. The survival map and Kaplan–Meier plots with significant results are displayed. The 95% confidence intervals of DFS are indicated by red and blue dotted lines for high and low DDX21 expression groups, respectively.

We separated cancer cases into high-expression and low-expression categories based on DDX21 expression levels and evaluated the relationship between DDX21 expression and prognosis in patients with various tumors, primarily utilizing TCGA and GEO databases. As shown in **Figure 3A**, high expression of DDX21 was linked to poor prognosis in terms of OS for ACC (**Figure 3B**, log-rank p = 0.00017), CESC (**Figure 3C**, log-rank p = 0.017), KIRP (**Figure 3E**, log-rank p = 0.037), MESO (**Figure 3F**, log-rank p = 0.001), and PAAD (**Figure 3G**, log-rank p = 0.0089) cancers based on TCGA data. DFS analysis data (**Figure 4A**) showed a correlation between high DDX21 expression and poor prognosis for TCGA cases of ACC (**Figure 4B**, log-rank p = 3.6e - 05) and BLAC (**Figure 4C**, log-rank p = 0.038). Furthermore, low DDX21 gene expression was associated with a poor OS prognosis for KIRC (**Figure 3D**, log-rank p = 0.0034) and a poor DFS prognosis for ESCA (**Figure 4D**, log-rank p = 0.028) and KIRC (**Figure 4E**, log-rank p = 0.046).

**Figure 5:**
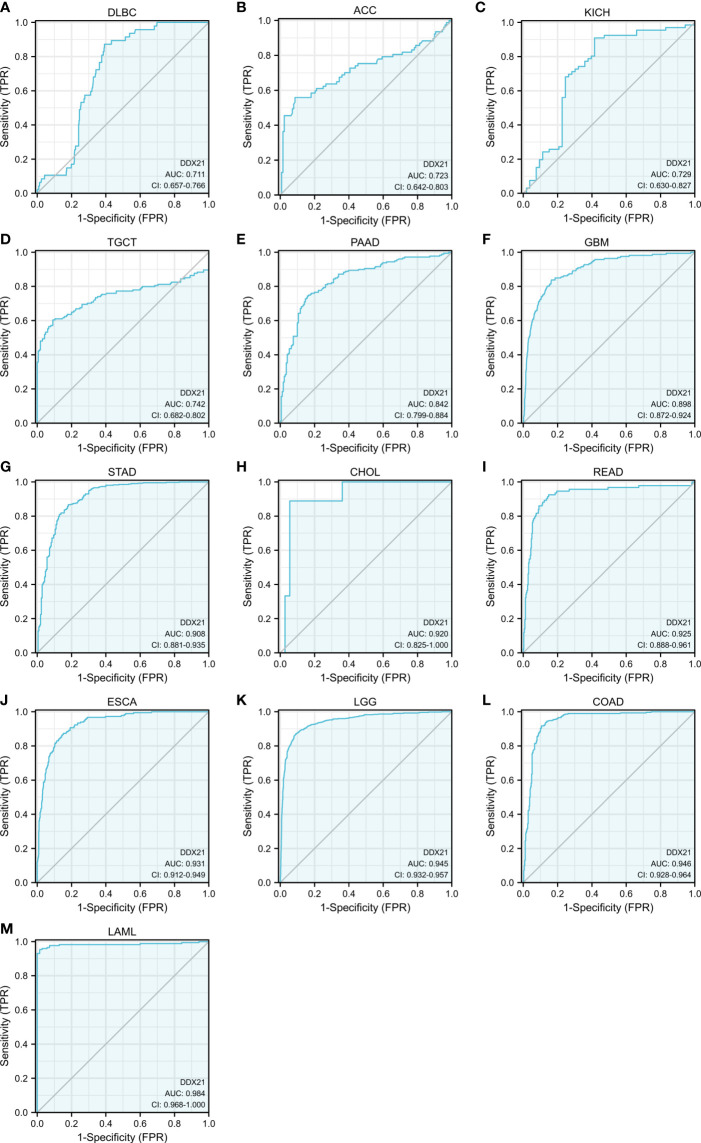
Analysis of receiver operating characteristics (ROC) curves between DDX21 and tumor prognosis based on data from TCGA and GTEx databases. **(A–M)** The area under the ROC curve (AUC) for lymphoid neoplasm diffuse large B-cell lymphoma (DLBC), adrenocortical carcinoma (ACC), kidney chromophobe (KICH), testicular germ cell tumors (TGCT), pancreatic adenocarcinoma (PAAD), glioblastoma multiforme (GBM), stomach adenocarcinoma (STAD), cholangiocarcinoma (CHOL), rectum adenocarcinoma (READ), esophageal carcinoma (ESCA), brain lower-grade glioma (LGG), colon adenocarcinoma (COAD), and acute myeloid leukemia (LAML) was 0.711, 0.723, 0.729, 0.742, 0.842, 0.898, 0.908, 0.920, 0.925, 0.931, 0.945, 0.946, and 0.984, respectively, indicating a high predictive value for tumor prognosis.

**Figures 5A–M** show that the AUC values for DLBC (AUC = 0.711), ACC (AUC = 0.723), KICH (AUC = 0.729), TGCT (AUC = 0.742), PAAD (AUC = 0.842), GBM (AUC = 0.898), STAD (AUC = 0.908), CHOL (AUC = 0.920), READ (AUC = 0.925), ESCA (AUC = 0.931), LGG (AUC = 0.945), COAD (AUC = 0.946), and LAML (AUC = 0.984) all exceeded 0.7, suggesting that DDX21 is a highly reliable predictor.

### Landscape of DDX21 mutation profiles in different tissues

**Figure 6:**
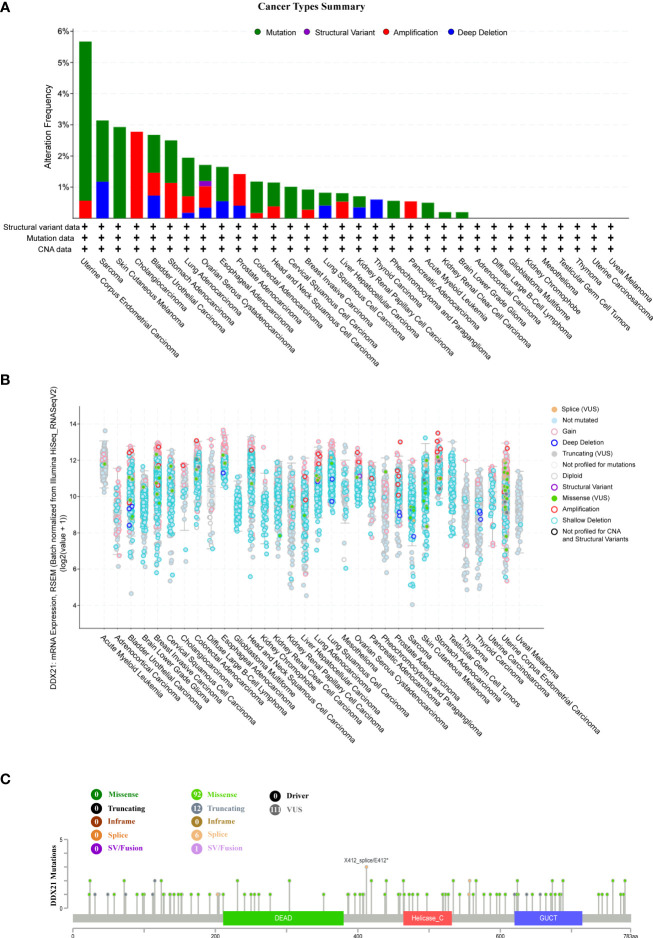
DDX21 mutation landscape. **(A)** DDX21 mutation frequency in multiple TCGA pan-cancer studies according to the cBioPortal database. **(B)** General mutation count of DDX21 in various TCGA cancer types according to the cBioPortal database. **(C)** Mutation diagram of DDX21 in different cancer types across protein domains.

We next used cBioPortal to investigate the DDX21 mutation frequency in TCGA database (10,967 samples in 32 studies) and discovered that UCEC shared a significantly high mutation level, with the DDX21 alteration frequency >5% (**Figures 6A, B**). Most of the genetic changes that occurred in UCEC tumor samples were copy number mutations (**Supplementary Table S2B**), which were the most common form of genetic alteration in all TCGA tumor samples. A total of 111 DDX21 mutations were identified, including 92 missense mutations, 12 truncating mutations, six splice mutations, and one sv/fusion mutation (**Supplementary Table S2C**; **Figure 6C**). Among them, X412_splice/E412* were the most frequent mutation sites.

### Immune infiltration analysis

**Figure 7:**
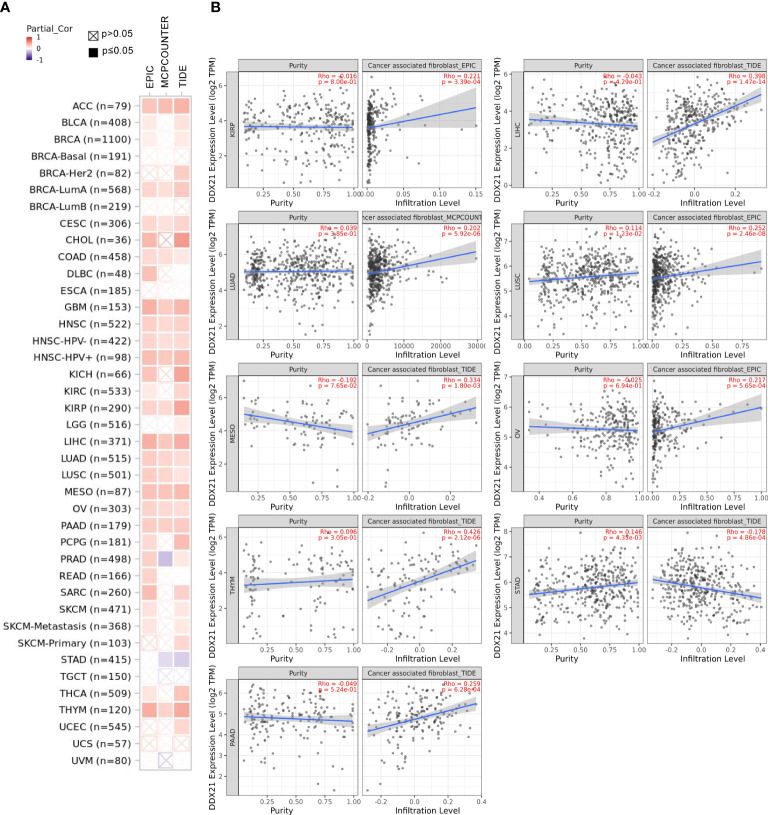
**(A, B)** Correlation between DDX21 expression and cancer-associated fibroblast immune infiltration. EPIC, MCPCOUNTER, and TIDE algorithms were used to calculate the correlation between DDX21 expression and cancer-associated fibroblast immune infiltration in all tumor types in TCGA.

Tumor-infiltrating immune cells, as critical parts of the tumor microenvironment, are closely related to cancer initiation, progression, and metastasis (16, 17). Cancer-associated fibroblasts in the stroma of the tumor microenvironment are thought to play a role in influencing the actions of various tumor-infiltrating immune cells (18, 19). In the present study, we employed the TIMER, CIBERSORT, CIBERSORT-ABS, QUANTISEQ, XCELL, MCPCOUNTER, and EPIC algorithms to explore the possible link between the infiltration level of different immune cells and DDX21 gene expression in various cancer types according to TCGA database. Based on most or all algorithms, we observed a statistically significant negative correlation between the immune infiltration of CD8^+^ T cells and DDX21 expression in BRCA-Her2, HNSC (human papillomavirus [HPV]^+/−^), THYM, and UCEC tumors (**Supplementary Figure S6**). Furthermore, we found a statistically significant positive correlation between DDX21 expression and the approximated infiltration value of cancer-associated fibroblasts for TCGA tumors ACC, BRCA-LumA, CESC, COAD, GBM, HNSC (HPV^+/−^), KIRP, LIHC, LUAD, LUSC, MESO, OV, PAAD, and THYM but noted a negative correlation for STAD (**Figure 7A**). **Figure 7B** and **Supplementary Figure S6** show scatterplot data for the aforementioned tumors generated by a single algorithm. According to the TIDE algorithm, the degree of DDX21 expression in STAD is negatively correlated with the level of infiltration of cancer-associated fibroblasts (**Figure 7B**, cor = -0.178, p = 4.86e-04).

### DDX21-related gene enrichment

**Figure 8:**
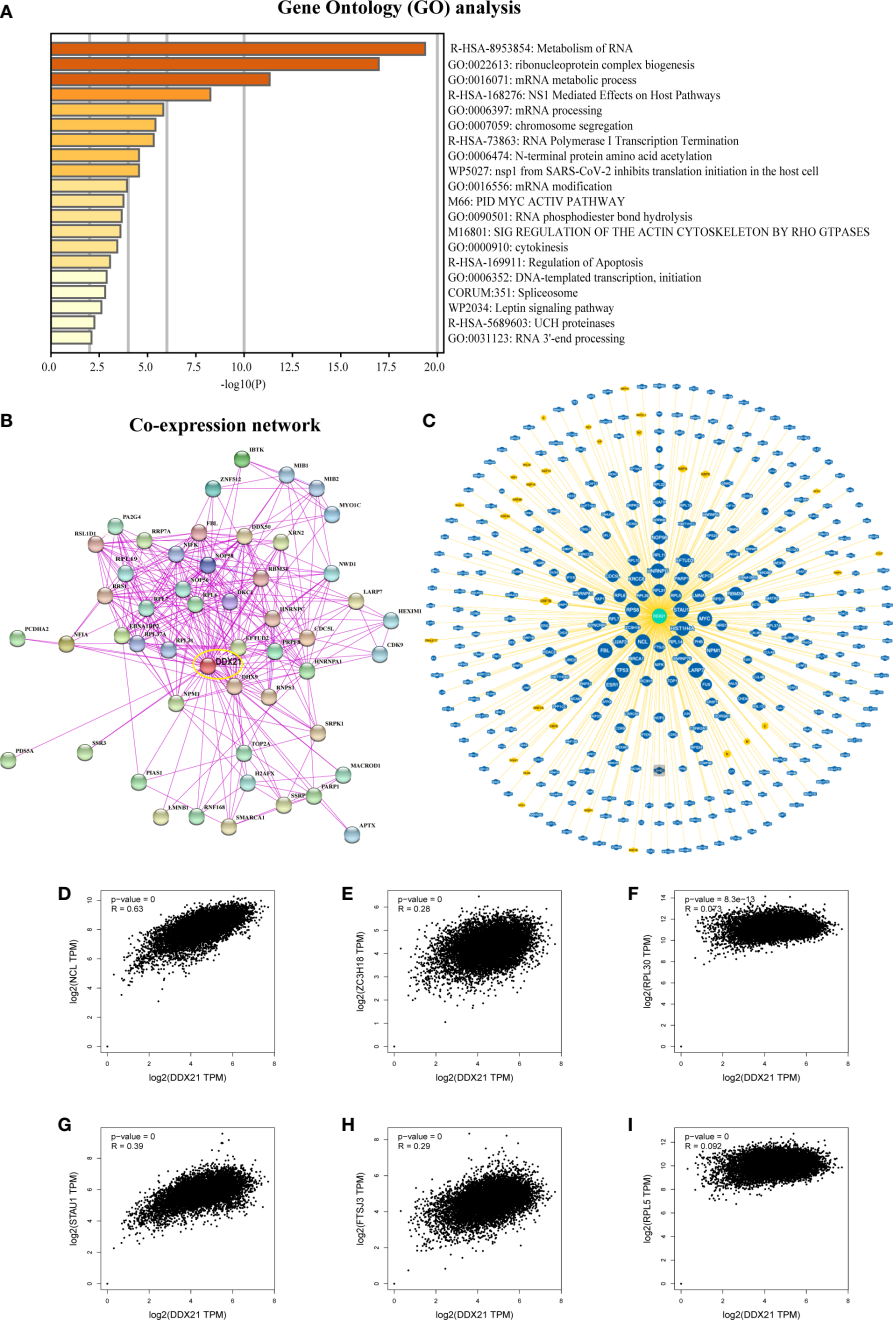
DDX21-related gene enrichment analysis. **(A)** Gene Ontology (GO) analysis of the top 100 genes co-expressed with DDX21 obtained by GEPIA2. **(B)** Co-expression network of 50 genes co-expressed with DDX21 obtained by the STRING tool. **(C)** DDX21–protein interactions obtained by BioGRID. **(D−I)** Correlation analysis between DDX21 and RPL5, RPL30, ZC3H18, NCL, FTSJ3, and STAU1 conducted by GEPIA2 across all tumor samples in TCGA.

To investigate the functional mechanism of DDX21 in the occurrence of cancer, we used GEPIA2 to identify the top 100 genes in TCGA dataset with expression patterns comparable with DDX21 (**Supplementary Table S2D**). Functional studies of these genes through the Metascape website showed that these genes are closely related to RNA metabolism or ribosomal protein synthesis (ribosomal biogenesis; **Figure 8A**). Subsequently, 50 proteins co-expressed with DDX21 were acquired using the STRING tool to verify the results of gene enrichment analysis. As shown in **Figure 8B**, correlations for these 50 genes are very strong; in addition, these genes are also implicated in regulatory pathways related to ribosome synthesis (**Supplementary Table S2E**). These findings compelled us to explore whether DDX21 participates in these biological processes by interacting with critical proteins involved in RNA metabolism and ribosomal protein synthesis. According to the BioGRID 4.3 database, DDX21 physically interacts with RPL5, RPL31, RPL30, RPL14, RPS6, ZC3H18, NCL, FTSJ3, HIST1H4A, and STAU1 (**Figure 8C**). These proteins function in RNA metabolism, protein synthesis, and tumorigenesis (**Supplementary Table S2F-2H**) (20–29). In addition, the expression of DDX21 is tightly connected to the expression levels of NCL, ZC3H18, RPL30, STAU1, FTSJ3, and RPL5 (**Figures 8D-I**). Based on these findings, we hypothesize that DDX21 may exert a tumor-promoting effect in cancer by driving RNA metabolism and promoting ribosomal protein synthesis.

### DDX21 promotes LUAD cell proliferation and metastasis *in vitro*

**Figure 9:**
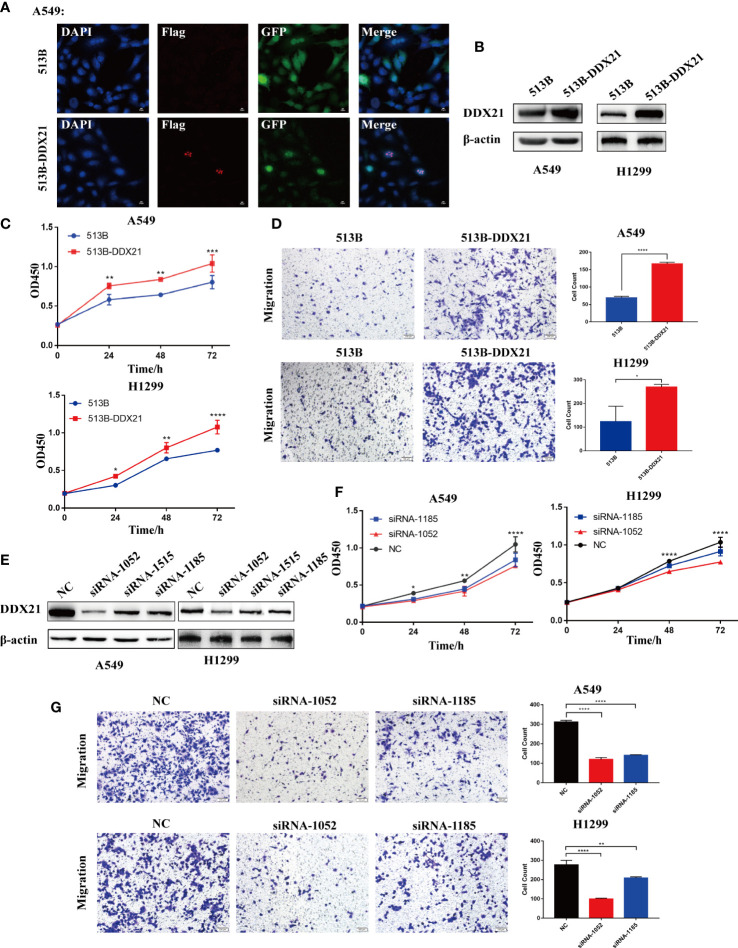
DDX10 promoted lung adenocarcinoma cancer cell growth and metastasis *in vitro*. **(A)** The localization of Flag-tagged DDX21 assayed by indirect immunofluorescence in DDX21 overexpression A549 cells. **(B–D)** A549 and H1299 cells were infected with lentivirus. Western blot was performed to detect the expression of DDX21; cell growth was detected by CCK-8 assays after 0, 24, 48, and 72 h (n = 3), and cell migration was evaluated by Transwell migration assay. **(E–G)** A549 and H1299 cells were transfected with siRNA target to DDX21, and cell proliferation and migration were evaluated by CCK-8 and Transwell migration assay, respectively. (*p < 0.05; **p < 0.01; ***p < 0.001, ****p < 0.0001).

To clarify the effect of DDX21 on the proliferation and migration of LUAD cells, we generated DDX21-overexpressing (513B-DDX21) and knockdown (siRNA-1052 and siRNA-1185) A549 and H1299 cells. DDX21 was mainly located in the nucleolus (**Figure 9A**), consistent with previous studies (30). DDX21 was overexpressed after lentivirus infection, and cell proliferation was dramatically boosted (**Figures 9B, C**). Meanwhile, cell migration was improved (**Figure 9D**). On the contrary, the siRNA significantly suppressed DDX21 expression in A549 and H1299 cells and markedly inhibited cell proliferation and migration (**Figures 9E-G**). These results demonstrated that DDX21 has the potential to enhance the growth and metastasis of LUAD cells, consistent with previous bioinformatics results.

## Discussion

DDX21, also known as nucleolar RNA helicase 2, is an autoantigen with autoantibodies detected in patients suffering from connective tissue diseases and gastric antral vascular ectasia (watermelon stomach disease) (31–33). DDX21 performs a variety of functions in ribosome biogenesis, including transcription and ribosomal RNA processing (34–37). DDX21 can effectively unwind R-loops (three-stranded nucleic acid complexes composed of an RNA: DNA heteroduplex) and prevent R-loop-mediated stalling of RNA polymerases, whereas DDX21 deficiency causes cellular R-loops to build up, resulting in DNA damage (5, 38). In the past decade, DDX21 has attracted attention due to its potential role in tumorigenesis. One study (39) found that DDX21 promotes the proliferation of gastric cancer by regulating the cell cycle. Another recent study found that downregulation of long non-coding RNA (lncRNA) HCP5 can limit the proliferation, migration, and invasion of gastric cancer cells by regulating the expression of DDX21 (40). Another study confirmed DDX21 as a prognostic marker for early colorectal cancer with microsatellite instability (41), which might be a new precision treatment strategy (42). Nevertheless, the role of DDX21 in different tumor types has not been thoroughly investigated. To this end, we herein analyzed the characteristics of DDX21 gene expression, protein expression, immunohistochemistry, gene mutation, immune infiltration, and protein phosphorylation, and the DDX21 genes of 33 TCGA tumor types were systematically identified.

We found that the DDX21 gene is significantly expressed in 13 cancers, and IHC analysis confirmed this trend at the protein level. Our results are consistent with previous reports that DDX21 is upregulated at gene and protein levels in colorectal cancer (41, 43, 44), breast cancer (8, 45), and lymphoma (46). We explored the link between DDX21 overexpression and clinical parameters or prognosis. Overexpression of DDX21 was related to poor OS and DFS in a survival study. In different tumor types such as ACC, CESC, KIRP, MESO, PAAD, and BLCA, high DDX21 expression is associated with poor prognosis.

Accumulating evidence indicates that genomic mutations are related to tumor progression and chemotherapy responses (34, 35). For example, Yang et al. (34) found that BRCA1 and BRCA2 mutations are strongly related to patient survival, which may be due to a significant response to platinum-based pharmacological treatment. A large-scale study discovered that mutations in four genes (ESR1, CDH1, RICTOR, and TP53) frequently occur in particular metastatic regions, suggesting that these genes might serve as biomarkers or therapeutic targets for patients with metastatic breast cancer (35). In the present study, we found that DDX21 mutations were the most common (>5%) in UCEC, followed by SARC, SKCM, CHOL, BLCA, and STAD. In summary, these findings suggest that DDX21 acts as an oncogene in the progression of a number of cancers and is a viable predictor of cancer prognosis in practical applications.

Immune cells are inextricably linked to cancer cells and have a considerable influence on cancer migration and metastasis in a variety of tumor forms (36). Recent studies reported that the tumor immune microenvironment is related to the expression levels of various genes (37, 47). In the present work, we found that DDX21 expression was positively correlated with CAF and CD8^+^ filtering in several tumor types, such as ACC, BRCA-LumA, CESC, COAD, GBM, HNSC (HPV^+/−^), KIRP, LIHC, LUAD, LUSC, MESO, OV, PAAD, and THYM. CAF is an important component of tumor cells and is reportedly correlated with a worse prognosis for various cancers, chemotherapy resistance, and disease recurrence (48–51). In summary, our work clarified the potential role of DDX21 in tumor immunity and its prognostic value for a variety of cancers.

Using STRING and GEPIA2, we discovered a plethora of genes that are co-expressed with DDX21 in various tumors. Metascape gene enrichment analysis showed that DDX21 is closely related to RNA metabolism or ribosomal protein production, consistent with previous studies (30). In addition, our results indicate that DDX21 physically interacts with RPL5, RPL31, RPL30, RPL14, RPS6, ZC3H18, NCL, FTSJ3, HIST1H4A, and STAU1. The expression of RPL5, RPL30, ZC3H18, NCL, FTSJ3, and STAU1 is closely related to the expression of DDX21. RPL5, RPL31, RPL30, RPL14, RPS6, ZC3H18, NCL, FTSJ3, HIST1H4A, and STAU1 are well-characterized genes encoding proteins involved in DNA repair and cell-cycle regulation (20–29). These discoveries validate the results of our gene enrichment study and open the path for further research into the molecular functions of DDX21.

In the previous gene and protein expression analysis, we found that the expression of DDX21 in LUAD was higher than that in normal tissues and associated with the advanced stages of LUAD. We hypothesize that DDX21 might be an oncogene. We verified the analysis results on the LUAD cell line through *in vitro* experiments. According to the results, it can be found that the expression of DDX21 in LUAD is proportional to the cell proliferation and migration ability.

However, the current study has some limitations. Firstly, the sample size for several rare tumor types was limited, which may have led to batch effects or erroneous results. Secondly, this research only presents early evidence associating DDX21 with cancer progression in a variety of tumors. The prediction results are not verified by experiments. Further research using methods such as reverse transcription PCR (RT-PCR) and immunocytochemistry should be performed to understand the specific molecular functions of DDX21 in carcinogenesis.

In summary, DDX21 is commonly overexpressed in a variety of cancers, and its gene expression is statistically linked with some clinical outcomes in some patients. Furthermore, immune infiltration and DDX21-related gene enrichment analyses suggest a possible mechanism by which DDX21 regulates tumor immunity, RNA metabolism, or ribosomal protein synthesis. Therefore, further experiments and clinical trials are needed to investigate the practical applicability of DDX21 in cancer treatment and prognosis prediction.

## Data availability statement

The raw data supporting the conclusions of this article will be made available by the authors, without undue reservation.

## Author contributions

Investigation and writing the original draft, YW. Methodology, JT, ZC and RC. Project administration, XH, YC, AH and TL. Funding acquisition, QC. All authors contributed to the article and approved the submitted version.

## Funding

This work was supported by the National Natural Science Foundation of China (Grant No. 32172827).

## Acknowledgments

All the experiments in this article were completed in the Laboratory Animal Center of Xuzhou Medical University. We thank the teachers for their support and help during the experiments.

## Conflict of interest

The authors declare that the research was conducted in the absence of any commercial or financial relationships that could be construed as a potential conflict of interest.

## Publisher’s note

All claims expressed in this article are solely those of the authors and do not necessarily represent those of their affiliated organizations, or those of the publisher, the editors and the reviewers. Any product that may be evaluated in this article, or claim that may be made by its manufacturer, is not guaranteed or endorsed by the publisher.

## Supplementary material

Supplementary Figure 1 Workflow diagrams for the methodology in this study as well as the workflow showing the steps including integration of datasets from various sources.

Supplementary Figure 2 Workflow diagrams for the methodology in this study as well as the workflow showing the steps including integration of datasets from various sources.

Supplementary Figure 3 Expression levels of the DDX21 gene in different tumors. **(A−F)** The expression status of the DDX21 gene in ACC, BRAC, OV, SARC, TGCT and UCS in TCGA were compared with corresponding normal tissues in GTEx databases.

Supplementary Figure 4 DDX21 expression status in different normal tissues. **(A−C)** DDX21 tissue expression based on human protein atlas (HPA), GTEx and function annotation of the mammalian genome 5 (FANTOM5) databases. **(D)** DDX21 expression in various cell types.

Supplementary Figure 5
**(A−M)** Single cell RNA levels of designated cell type clusters for each examined tissue. The bar chart depicts the amounts of nTPM in each annotated cluster of single cells. The UMAP plot depicts the RNA expression profile on the left. Color coding is based on cell type groupings, which are made up of cell types that share functional characteristics. Note that the same cell type may be present in multiple clusters.

Supplementary Figure 6
**(A, B)** Correlation analysis between DDX21 expression and immune infiltration of CD8^+^ T-cells. Different algorithms were used to explore the potential correlation between the expression level of the DDX21 gene and the infiltration level of CD8^+^ T-cells across all types of cancer in TCGA.
