# Supplementary figures and images for: Pan-cancer analysis reveals DDX21 as a potential biomarker for the prognosis of multiple tumor types
Source: Front Oncol. 2022 Nov 24;12:947054. doi: 10.3389/fonc.2022.947054 (PMC9730287; doi:10.3389/fonc.2022.947054)

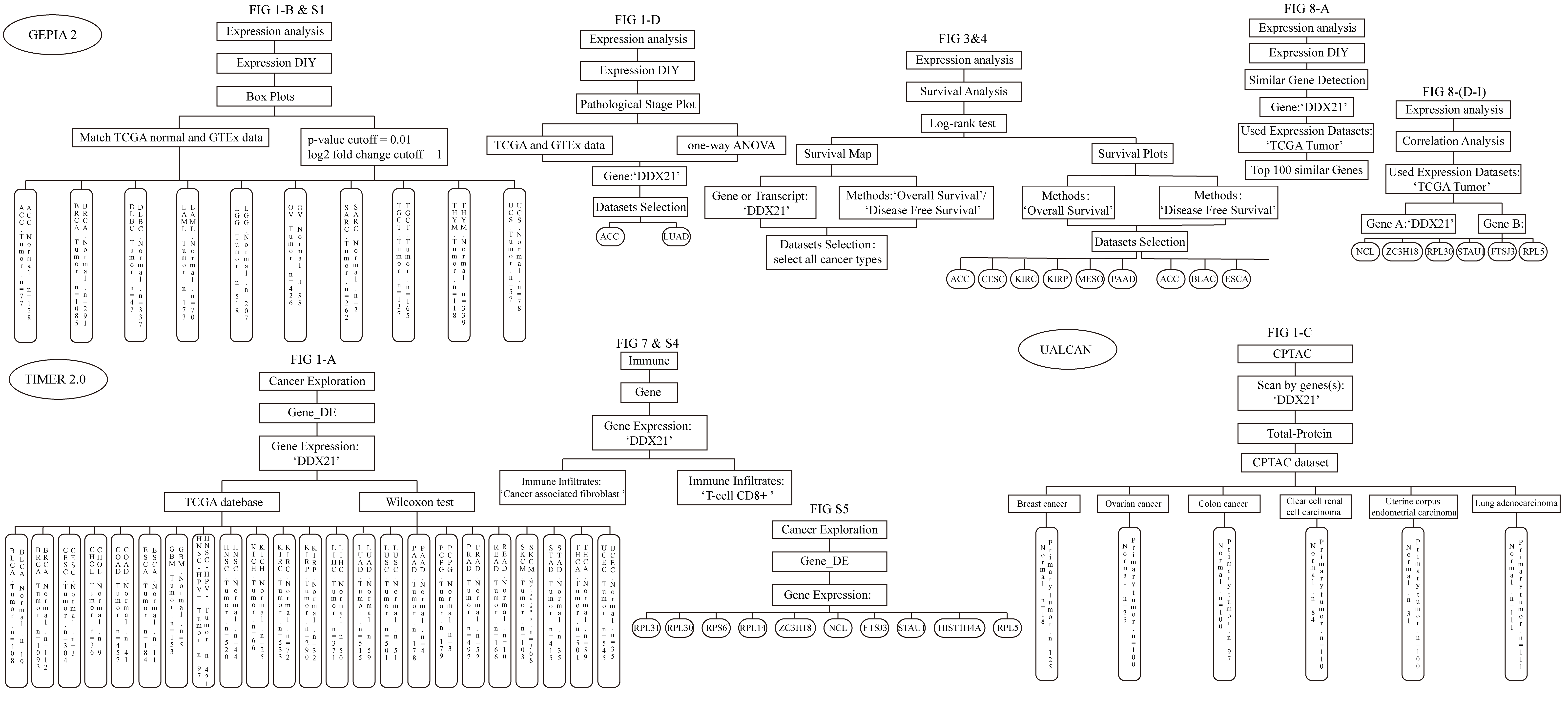

Supplement: Supplementary Figure 1 — Workflow diagrams for the methodology in this study as well as the workflow showing the steps including integration of datasets from various sources. [file Image_1.tif]

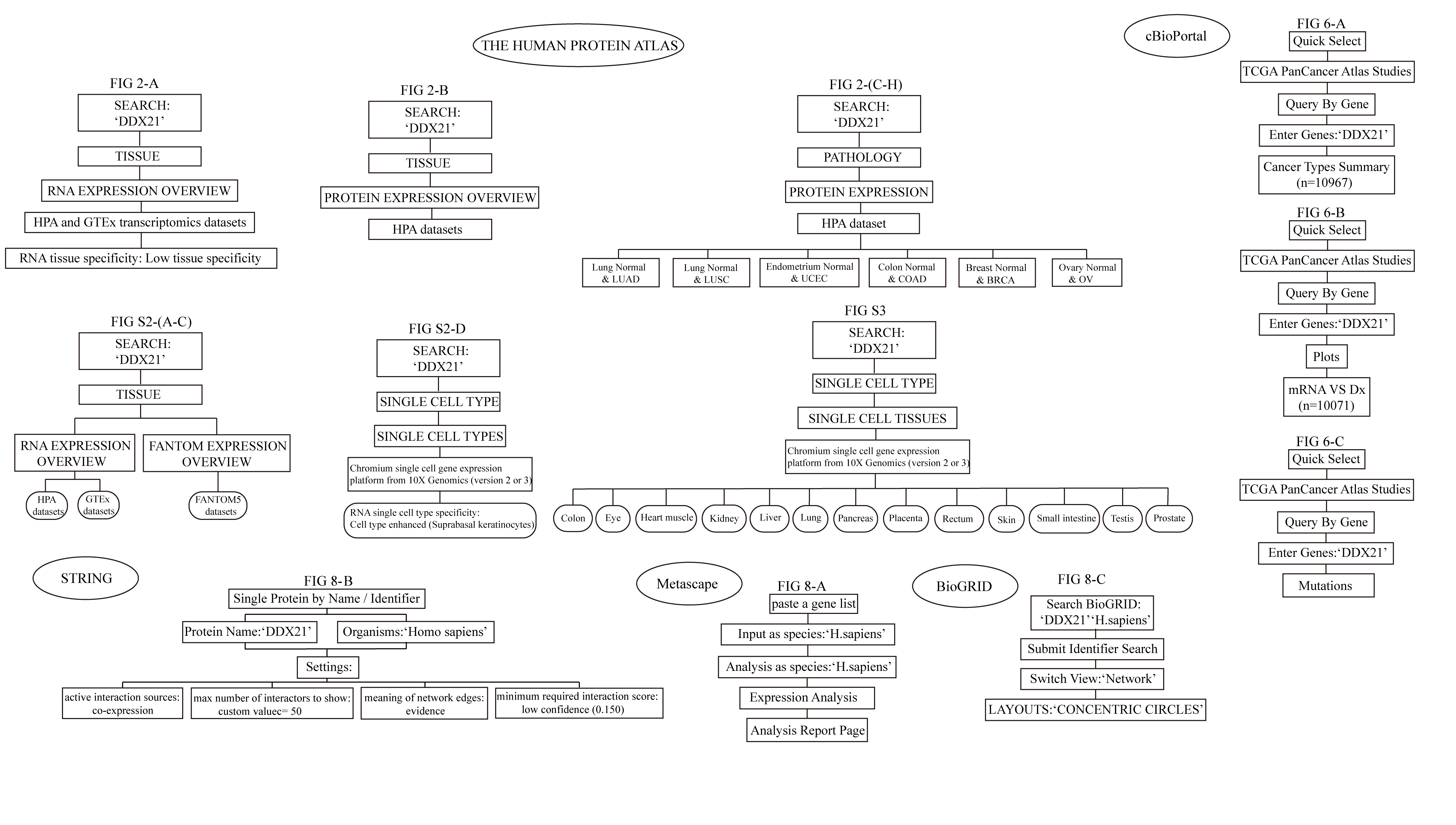

Supplement: Supplementary Figure 2 — Workflow diagrams for the methodology in this study as well as the workflow showing the steps including integration of datasets from various sources. [file Image_2.tif]

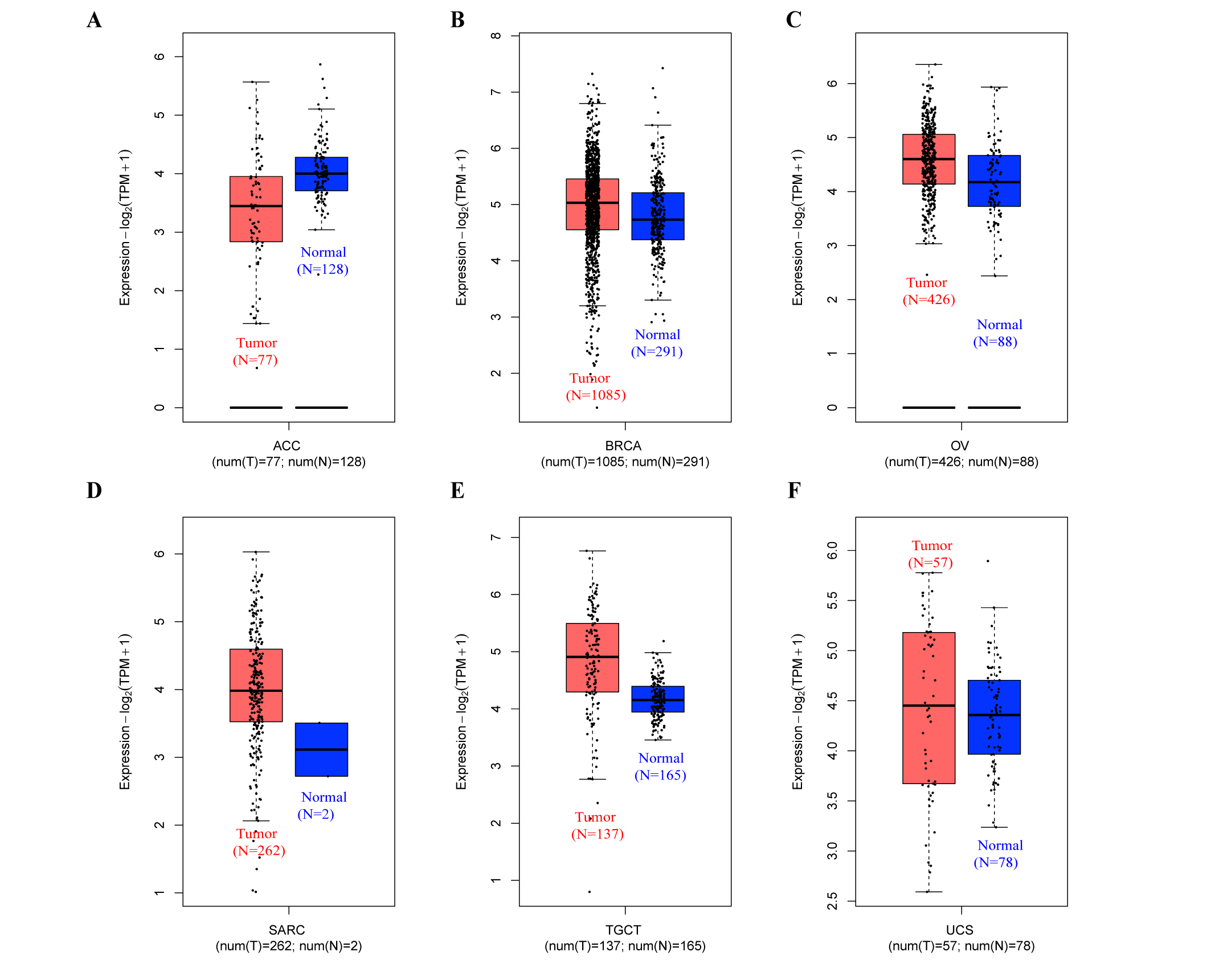

Supplement: Supplementary Figure 3 — Expression levels of the DDX21 gene in different tumors. (A−F) The expression status of the DDX21 gene in ACC, BRAC, OV, SARC, TGCT and UCS in TCGA were compared with corresponding normal tissues in GTEx databases. [file Image_3.tif]

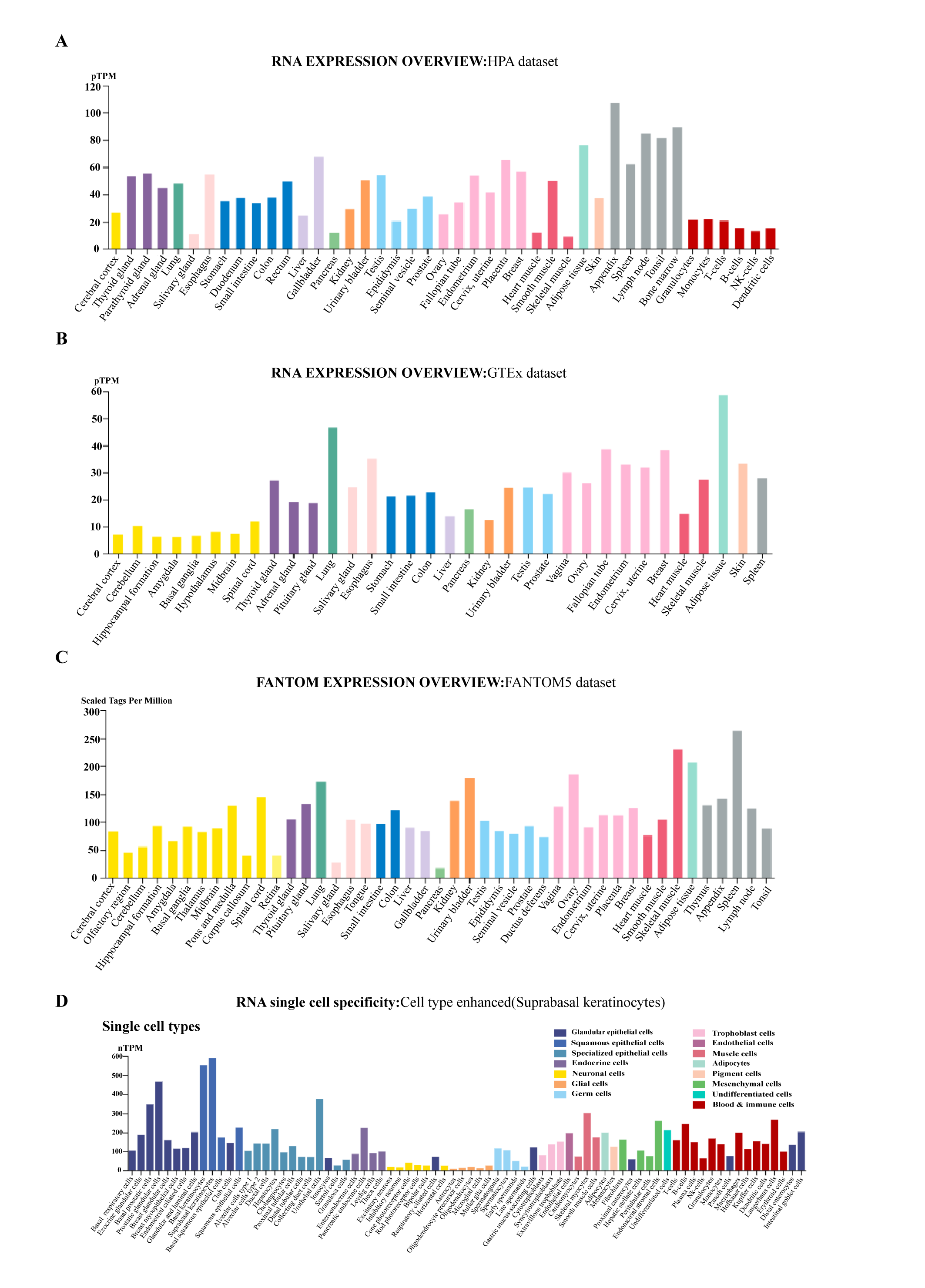

Supplement: Supplementary Figure 4 — DDX21 expression status in different normal tissues. (A−C) DDX21 tissue expression based on human protein atlas (HPA), GTEx and function annotation of the mammalian genome 5 (FANTOM5) databases. (D) DDX21 expression in various cell types. [file Image_4.tif]

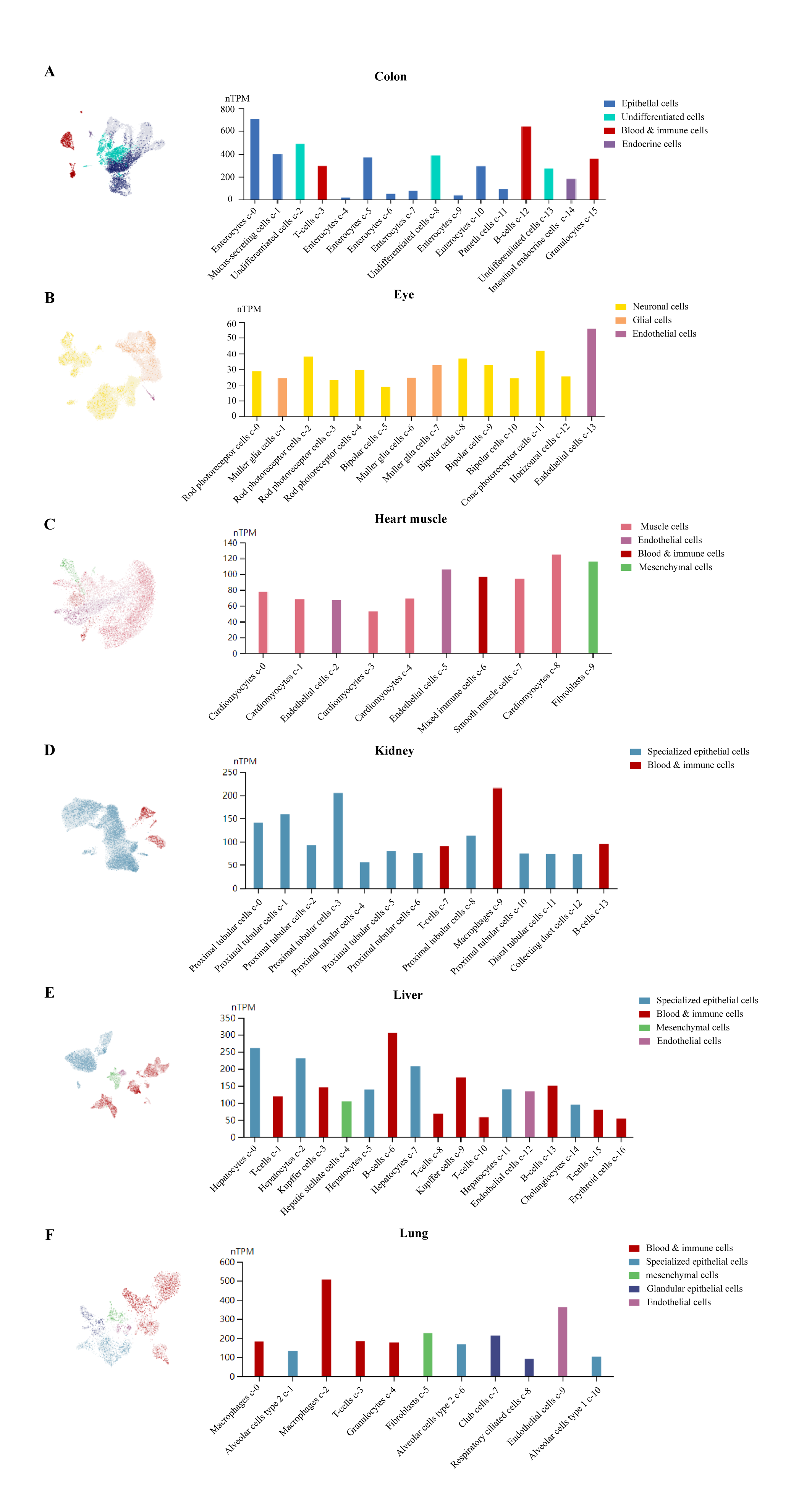

Supplement: Supplementary Figure 5 — (A−M) Single cell RNA levels of designated cell type clusters for each examined tissue. The bar chart depicts the amounts of nTPM in each annotated cluster of single cells. The UMAP plot depicts the RNA expression profile on the left. Color coding is based on cell type groupings, which are made up of cell types that share functional characteristics. Note that the same cell type may be present in multiple clusters. [file Image_5.tif]

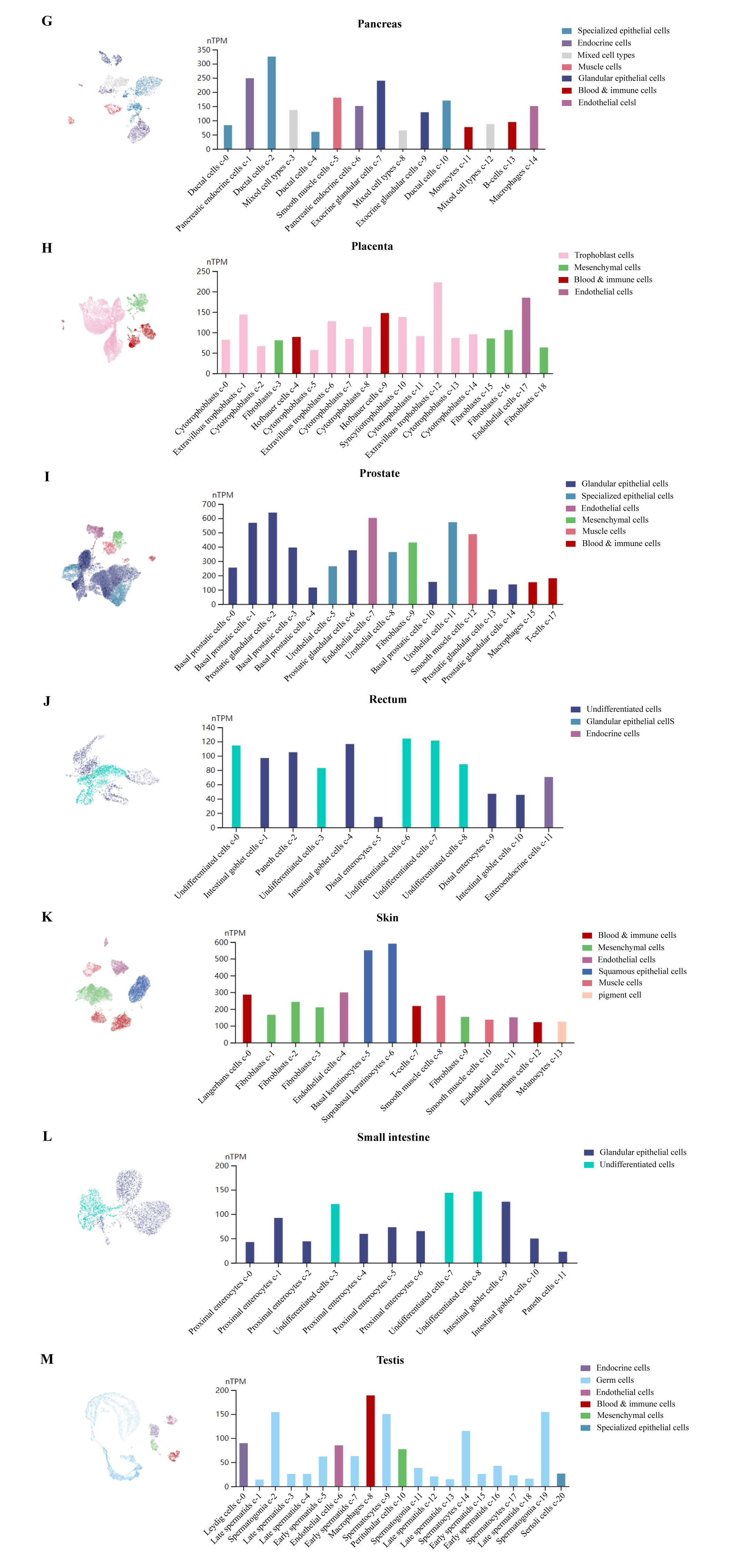

Supplement: Supplementary Figure 6 — (A, B) Correlation analysis between DDX21 expression and immune infiltration of CD8+ T-cells. Different algorithms were used to explore the potential correlation between the expression level of the DDX21 gene and the infiltration level of CD8+ T-cells across all types of cancer in TCGA. [file Image_6.tif]

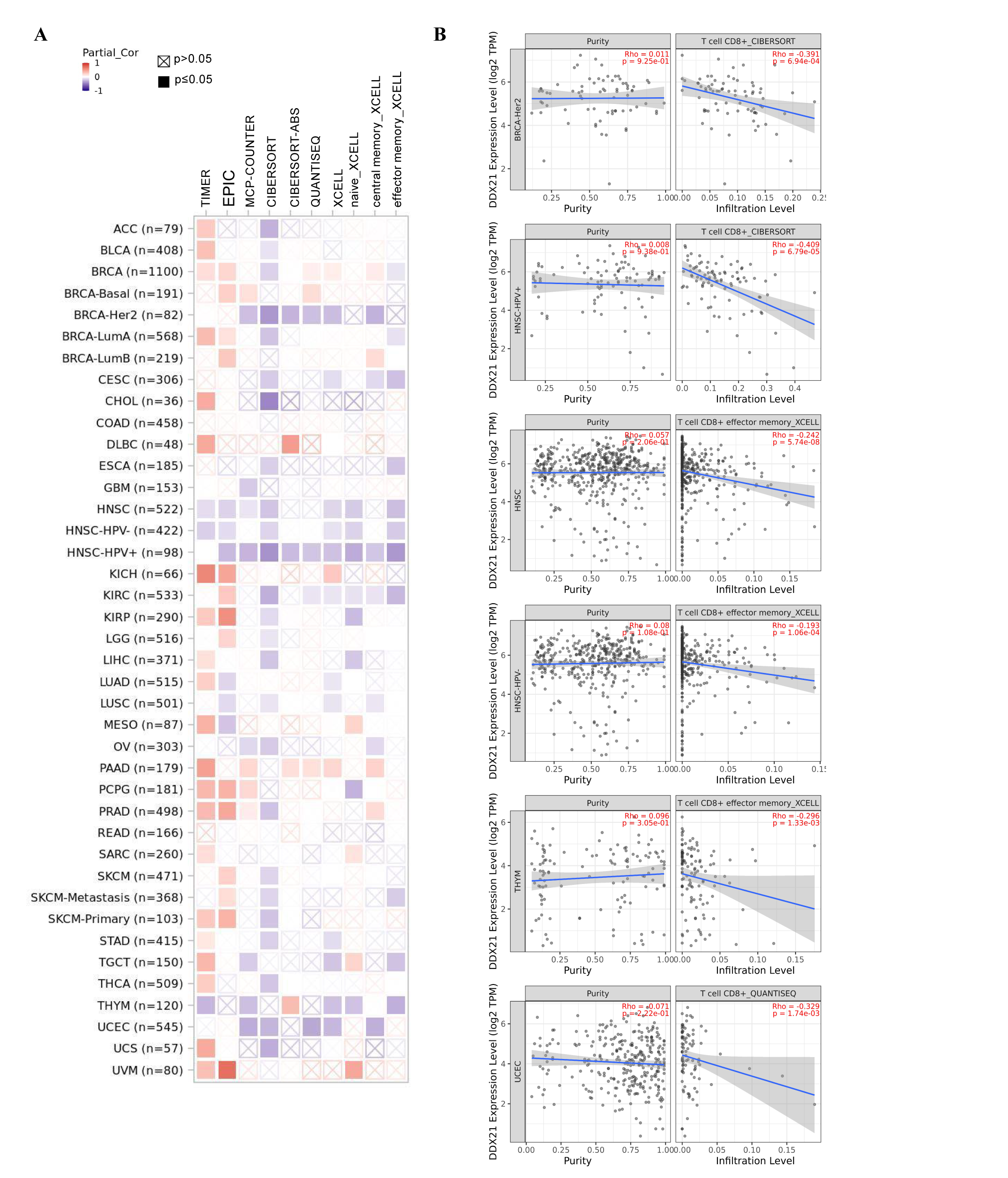

Supplement: Supplementary file 7 [file Image_7.tif]
